# Transgenic switchgrass (*Panicum virgatum* L.) targeted for reduced recalcitrance to bioconversion: a 2‐year comparative analysis of field‐grown lines modified for target gene or genetic element expression

**DOI:** 10.1111/pbi.12666

**Published:** 2017-02-20

**Authors:** Alexandru Dumitrache, Jace Natzke, Miguel Rodriguez, Kelsey L. Yee, Olivia A. Thompson, Charleson R. Poovaiah, Hui Shen, Mitra Mazarei, Holly L. Baxter, Chunxiang Fu, Zeng‐Yu Wang, Ajaya K. Biswal, Guifen Li, Avinash C. Srivastava, Yuhong Tang, Charles Neal Stewart, Richard A. Dixon, Richard S. Nelson, Debra Mohnen, Jonathan Mielenz, Steven D. Brown, Brian H. Davison

**Affiliations:** ^1^ BioEnergy Science CenterOak Ridge National LaboratoryOak RidgeTNUSA; ^2^ Biosciences DivisionOak Ridge National LaboratoryOak RidgeTNUSA; ^3^ Department of Plant SciencesUniversity of TennesseeKnoxvilleTNUSA; ^4^ BioDiscovery Institute and Department of Biological SciencesUniversity of North TexasDentonTXUSA; ^5^ Forage Improvement DivisionThe Samuel Roberts Noble FoundationArdmoreOKUSA; ^6^ Complex Carbohydrate Research Center and Department of Biochemistry and Molecular BiologyUniversity of GeorgiaAthensGAUSA; ^7^ Plant Biology DivisionThe Samuel Roberts Noble FoundationArdmoreOKUSA

**Keywords:** bioconversion, bioenergy, recalcitrance, switchgrass, transgenic, comparison

## Abstract

Transgenic *Panicum virgatum* L. silencing (KD) or overexpressing (OE) specific genes or a small RNA (*GAUT4*‐KD, miRNA156‐OE, MYB4‐OE,*COMT*‐KD and *FPGS*‐KD) was grown in the field and aerial tissue analysed for biofuel production traits. Clones representing independent transgenic lines were established and senesced tissue was sampled after year 1 and 2 growth cycles. Biomass was analysed for wall sugars, recalcitrance to enzymatic digestibility and biofuel production using separate hydrolysis and fermentation. No correlation was found between plant carbohydrate content and biofuel production pointing to overriding structural and compositional elements that influence recalcitrance. Biomass yields were greater for all lines in the second year as plants establish in the field and standard amounts of biomass analysed from each line had more glucan, xylan and less ethanol (g/g basis) in the second‐ versus the first‐year samples, pointing to a broad increase in tissue recalcitrance after regrowth from the perennial root. However, biomass from second‐year growth of transgenics targeted for wall modification, *GAUT4*‐KD,*MYB4*‐OE,*COMT*‐KD and *FPGS*‐KD, had increased carbohydrate and ethanol yields (up to 12% and 21%, respectively) compared with control samples. The parental plant lines were found to have a significant impact on recalcitrance which can be exploited in future strategies. This summarizes progress towards generating next‐generation bio‐feedstocks with improved properties for microbial and enzymatic deconstruction, while providing a comprehensive quantitative analysis for the bioconversion of multiple plant lines in five transgenic strategies.

## Introduction

Biomass is the primary renewable source of carbon for fuels and other carbon‐based products. However, it is economically challenging to convert lignocellulosic biomass to fuels. The inherent recalcitrance of biomass to sugar depolymerization is largely responsible for the high industrial cost of lignocellulose bioconversion (Himmel *et al*., 2007). Recalcitrance can be defined as ‘the natural resistance of plant cell walls to microbial and enzymatic deconstruction’ (Himmel *et al*., 2007) due to its complex carbohydrate‐ and lignin‐based structure. Biotechnology can improve this process through the development of better microbial or enzymatic conversion agents as well as by modifying the bio‐feedstock source itself (Biswal *et al*., 2015; Bryan *et al*., 2016; Davison *et al*., 2015; Mottiar *et al*., 2016; Wuddineh *et al*., 2015).

Switchgrass is a dedicated perennial herbaceous feedstock that is attractive for the production of biofuels and specialty chemicals (Keshwani and Cheng, 2009; Kwit *et al*., 2014). It is a native perennial plant with good yields (3‐8 tons/acre) and can be grown in a range of North American ecosystems (U.S. Department of Energy *et al*., 2016). It has increasing genetic resources (JGI Phytozyme 2016) and the strong potential to be grown with low inputs (McLaughlin and Adams Kszos, 2005; U.S. Department of Energy – Office of Biological and Environmental Research 2014). Understanding factors that influence recalcitrance is critical to improve switchgrass and other feedstocks as bioenergy fuel sources and multiple cell wall biosynthesis gene targets for improving plants via breeding and biotechnology have been discussed (Jung *et al*., 2012).

Within the BioEnergy Science Center (BESC), reverse genetics experiments have been conducted to achieve modified expression of distinct and diverse cell wall biosynthetic genes in switchgrass with the goal of reducing recalcitrance. Transgenic switchgrass modifying the expression of various synthesis genes or gene regulator elements were grown in the glasshouse and screened for differences in enzymatic sugar release using a high‐throughput procedure (Decker *et al*., 2009). Transgenic lines with high sugar release and normal or better growth phenotype than the wild‐type and vector controls were selected for field‐based experiments in Knoxville, Tenn, USA.

Field‐grown switchgrass transgenic lines with high sugar release or good growth phenotypes included those silencing or OE: (i) GAUT4, *GAUT4*‐knockdown (KD) lines down‐regulating expression of *galacturonosyltransferase4*, a gene encoding an enzyme involved in pectin biosynthesis (Biswal *et al*., Biswal, A.K., Atmodjo, M.A., Li, M., Yoo, C.G., Pu, Y., Lee, Y.‐C., Zhang, J.Y., Bray, A., King, Z., LaFayette, P., Mohanty, S.S., Ryno, D., Yee, K., Thompson, O.A., Rodriguez Jr, M., Winkeler, K., Collins, C., Yang, X., Tan, L., Sykes, R.W., Gjersing, E., Ziebell, A., Turner, G.B., Decker, S.R., Parrot, W., Udvardi, M.K., Mielenz, J., Davis, M.F., Nelson, R.S., Ragauskas, A.J., and Mohnen, D.); (ii) miRNA, *miRNA156*‐overexpression (OE) lines OE miRNA156, a regulator of plant developmental processes (Fu *et al*., 2012); (iii) MYB4, *MYB4*‐OE lines OE PvMYB4, an R2R3‐type MYB repressor of the lignin biosynthetic pathway (Shen *et al*., 2012, 2013); (iv) COMT, *COMT*‐KD lines down‐regulating expression of caffeic acid *O*‐methyltransferase, a lignin biosynthetic gene (Fu *et al*., 2011); and (v) FPGS, *FPGS*‐KD lines down‐regulating expression of *folylpolyglutamate synthase 1*, a gene encoding a C1 metabolism enzyme believed to provide methyl groups for lignin biosynthesis (Srivastava *et al*., 2015).

In this study, clones of plants representing independent transgenic events and their respective nontransgenic control lines were investigated for biomass yield, carbohydrate composition and recalcitrance to bioconversion via separate hydrolysis and fermentation (SHF) to yield ethanol. Year‐over‐year changes in the composition and recalcitrance of the field grown biomass are discussed, and the improvements of the transgenic plants over the control lines for various traits are presented. Additionally, factors that influence bioconversion are identified and quantitatively compared. Absolute ethanol yields that can be obtained from switchgrass lines are not the focus or a targeted milestone of the study. These analyses serve as a foundation for comparing future modified switchgrass lines and provide insights for understanding factors influencing biomass recalcitrance.

## Results and discussion

In the current study, each gene or gene regulatory element is represented by clones from two independent transgenic events, labelled V1 and V2, and a control line (CT) linked genetically with the specific transgenic events, but not expressing the transgene (i.e., either clones from the parent plant used as explant source, parent seed used as explant source or seed segregating away from the transgene in the T1 generation). Clones representing the V1 line for each target gene or gene regulatory element were given the V1 designation because they had higher ethanol yields in bioconversion assays of second‐year growth tissue compared with tissue from clones representing the second independent transgenic line for that gene or gene regulatory element. Table S1 provides detailed information on sample replication and plant nomenclature used in the primary publications of each transgenic line. The parental lineages for each transgenic experiment originated from different sources and were processed independently by different researchers; therefore, variation among control lines was to be expected.

The transgenic lines studied in this paper were generated in the BESC with the aim of creating plants with reduced recalcitrance to biological deconstruction by enzymes and to understand the mechanisms that impart recalcitrance in plant biomass through modifications or disruptions of plant biosynthetic and regulatory networks. Plant biomass recalcitrance was tested through a standardized bioconversion assay, in which sugar release from non‐pretreated plant tissue by hydrolytic enzymatic mixtures was quantified along with subsequent fermentation to ethanol by a model yeast strain, *Saccharomyces cerevisiae* D5A. The recalcitrance assay used here to compare control biomass to biomass from transgenic lines modified in specific cell wall biosynthetic genes utilized non‐pretreated senesced switchgrass to accentuate differences in plant recalcitrance (with a cellulase activity of 24 FPU/g glucan). As the assay was not developed to fully hydrolyse cellulose, enzymatically derived glucose yields from biomass generally ranged from 15% to 40% of the total available glucose. Paye *et al*. (2016) observed 12%–20% solubilization for a yeast‐based simultaneous saccharification and fermentation for senesced Alamo switchgrass. These values compare to a carbohydrate solubilization of pretreated senesced switchgrass tissue of about 20% by a modified dilute acid pretreatment and about 80% when combined with an enzyme cocktail (Wolfrum *et al*., 2013) which is more comparable to current proposed industrial processes. The latter study reported combined glucose and xylose release from switchgrass of up to 0.6 g sugars/g biomass, and considering the theoretical stoichiometric conversion of these carbohydrates to ethanol at 0.51 g ethanol/g sugar, the maximum attainable ethanol yield in switchgrass can be estimated at 300 g ethanol/kg switchgrass. Repeating the calculation for glucose release alone, reported at a maximum of ~0.47 g glucose/g biomass, the achievable theoretical yield from glucose conversion alone is estimated at 240 g ethanol/kg switchgrass. By comparison, in the current study which utilized an assay screen that converted glucose to ethanol, where conversion was not aided by biomass pretreatment, and where the goal was to investigate recalcitrance changes due to genetic modifications and not to maximize bioconversion yields, the highest ethanol yield obtained was 56 g ethanol/kg switchgrass.

### Variations in plant glucan and xylan content

Recalcitrance to biological degradation can be significantly reduced through chemical and physical pretreatment processes which typically remove most of the lignin and/or the hemicelluloses, reduce cellulose crystallinity and increase the porosity of biomass (Brodeur *et al*., 2011). A recent review (Wyman *et al*., 2013) reported the release of up to 91% of combined glucose and xylose content in pretreated and then enzymatically hydrolysed switchgrass. Therefore, when bioconversions are coupled with strong pretreatment strategies, although high carbohydrate content may be recovered, the severe pretreatment may mask improved plant characteristics that could yield reduced recalcitrance, that is higher sugar yield, achievable under less severe pretreatment strategies from the improved plants. The goal here was to generate plants with reduced recalcitrance at lower pretreatment severities as a potential means to yield higher cost benefits.

**Figure 1:**
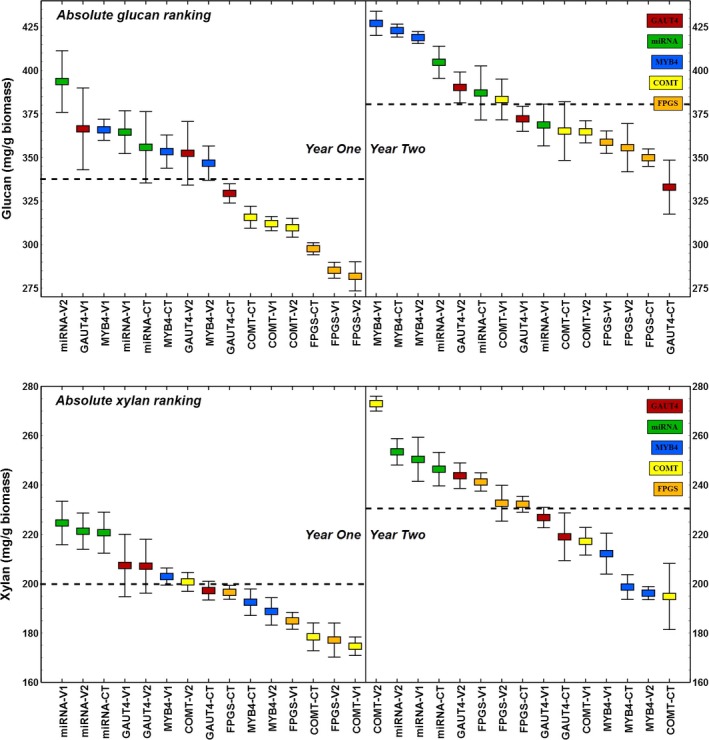
Decreasing rank of glucan and xylan abundance in all switchgrass lines separated by year of growth. These plant carbohydrates increased per gram biomass after regrowth for all plants. Horizontal dotted lines represent yearly averages and are significantly different at 95% confidence level.

Typical glucan content in switchgrass is 30%–45% of the cell wall biomass while xylan ranges from 20% to 25% (David and Ragauskas, 2010). Field‐grown switchgrass tiller cell walls in this study contained 28%–39% glucan in year one plants (avg., 34%), while year 2 plants showed a generalized increase in glucan content to 33%–43% (*P* < 0.001), with an average of 38% (Figure 1). On closer inspection, the growth year differences in glucan abundance were line specific, predominantly high in the COMT, MYB4 and FPGS lines and their controls and decidedly smaller or insignificant in the GAUT4 and miRNA lines and their controls (Table 1). The xylan content of year one plants spanned 17%–22% (average of 20%), which increased across all plants regrown in the field to 19%–27%, with an average of 23% (*P* < 0.001; Figure 1). A particularly modest increase in xylan content, up to 4.6%, was observed for the MYB4 transgenic lines while a very large change, up to 36%, was observed for the COMT transgenic lines (Table 1). The year‐over‐year changes in the xylan content of each line were statistically significant at 95% confidence level.

**Table 1:** Year‐over‐year changes in carbohydrate content and ethanol yield (as an indicator of tissue recalcitrance). Plant tissue increased in glucan and xylan abundance after regrowth in the field from belowground biomass. Uncorrelated to carbohydrate changes, tissue recalcitrance increased significantly (shown as lowered bioconversion to ethanol) for transgenes and parental plants alike in year two compared with year one. The year‐over‐year changes were significant at 95% confidence level with the exceptions marked with N/S superscript

	Glucan content (mg/g biomass) and year‐to‐year change (%)			Xylan content (mg/g biomass) and year‐to‐year change (%)			Ethanol yield (mg/g glucan) and year‐to‐year change (%)		
	Year 1	Year 2	Change %	Year 1	Year 2	Change%	Year 1	Year 2	Change %
GAUT4‐V1	366 (23)	372 (7)	^N/S^1.6	207 (12)	227 (4)	9.4	162 (8)	135 (3)	−16.4
GAUT4‐V2	352 (18)	390 (9)	10.7	207 (11)	244 (5)	17.7	143 (13)	103 (19)	−27.8
GAUT4‐CT	329 (6)	333 (15)	^N/S^1.1	197 (4)	219 (10)	11.1	146 (7.3)	131 (2)	−10.3
miRNA‐V1	365 (13)	369 (12)	^N/S^1.1	225 (9)	250 (9)	11.5	130 (12)	78 (17)	−40.2
miRNA‐V2	394 (18)	405 (9)	^N/S^2.8	221 (7)	253 (5)	14.5	121 (4)	75 (7)	−38.0
miRNA‐CT	356 (20)	387 (15)	8.8	221 (8)	246 (7)	−2.2	114 (3)	89 (17)	−22.0
MYB4‐V1	366 (6)	427 (7)	16.7	203 (3)	212 (8)	4.6	134 (8)	93 (7)	−30.8
MYB4‐V2	347 (10)	419 (3)	20.8	189 (6)	196 (3)	3.9	116 (5)	77 (6)	−33.0
MYB4‐CT	353 (10)	423 (4)	19.7	192 (5)	199 (5)	3.2	98 (3)	79 (5)	−20.2
COMT‐V1	312 (4)	383 (12)	22.8	175 (4)	217 (6)	24.4	149 (11)	84 (7)	−43.6
COMT‐V2	310 (5)	365 (6)	17.8	201 (4)	273 (3)	36.0	114 (13)	59 (2)	−48.7
COMT‐CT	316 (6)	365 (17)	15.7	178 (6)	195 (13)	9.2	120 (5)	69 (15)	−42.3
FPGS‐V1	285 (5)	359 (6)	25.8	185 (3)	241 (4)	30.4	179 (5)	83 (4)	−53.5
FPGS‐V2	282 (8)	356 (14)	26.2	177 (7)	233 (7)	31.3	169 (3)	70 (10)	−58.4
FPGS‐CT	298 (3)	350 (5)	17.6	196 (3)	232 (3)	18.7	161 (6)	73 (2)	−54.8

In the third and, where available, the fourth year of growth, changes in carbohydrate content were decidedly smaller, although still statistically significant. Comparing year 2 with year 3 results in all the transgenic and control plant lines used in this study, glucan varied in nine of 12 lines, while xylan varied in all 12 at 95% confidence level (original data not shown). To exemplify, glucan in the COMT‐V1 and COMT‐CT lines was shown to stabilize through years 2 to 4 with much smaller variations compared with the large change from the first to the second year (Figure S1). These observations suggest that tissue from year 3 and 4 transgenic and control plant seasons should have similar characteristics with tissue from the year 2 season.

Without pretreatment, enzymatic deconstruction of switchgrass biomass with glucan bioconversion to ethanol was utilized as a collective indicator of plant tissue recalcitrance. In this study, an insubstantial monotonic Spearman correlation (−0.36 coefficient, *P* < 0.001) between glucan content (mg/g biomass) and ethanol yield (mg/g biomass) was obtained when comparing 87 plot samples (Figure S2). Xylan content (mg/g biomass) also did not correlate with ethanol yields (−0.29 Spearman coefficient, *P* = 0.006). The systematic year‐over‐year changes in ethanol yields, discussed in detail in the following section, did not correlate with yearly increases in plant tissue glucan abundance (0.28 Spearman coefficient, *P* = 0.09) or in any substantial way with changes in xylan abundance (−0.36 Spearman, *P* = 0.02). These findings showed that plant recalcitrance was not affected by the abundance of primary carbohydrates, as determined in our sugar analysis assays (Figure S2), and higher sugar content did not translate to better bioconversion of non‐pretreated plant tissue. Rather, the results suggest that the reduced recalcitrance in the transgenic biomass discussed below, was due to cell wall structural changes.

**Figure 2:**
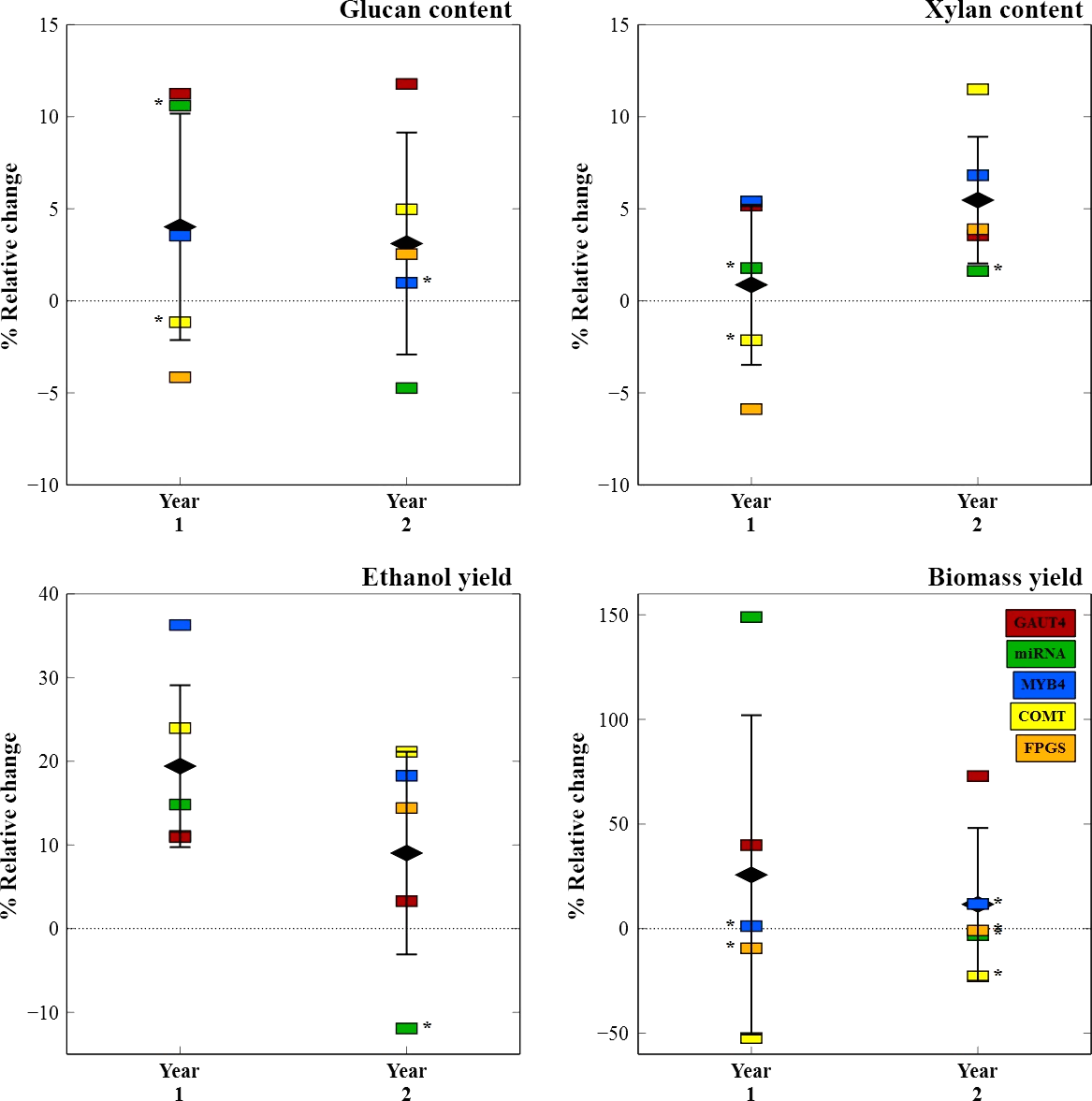
Relative changes in polymer sugars, ethanol yield and biomass yield from parental plant to best transgene line (i.e. CT to V1) for each switchgrass line. For the mature year 2 plants, V1 transgenic lines showed increased glucan and xylan abundances (mg/g biomass) and lower recalcitrance through higher ethanol yields (mg/g glucan) than their respective controls. The miRNA‐V1 plants are the exception to this general observation. FPGS year 1 ethanol yield value overlaps with GAUT4 and is not visible on the plot.

Alterations in glucan and xylan abundance due to genetic manipulations were also investigated. Although the genes targeted in the transgenic lines studied here were not expected to directly cause changes in cellulose content in the plant tissue, the majority of V1 transgenic variants showed small but significant differences in glucan from their parental controls (Figure 2, top left). The GAUT4‐V1 line, in particular with disrupted pectin biosynthesis, sustained glucan gains of 11% and 12% in each year, respectively. Disrupted lignin biosynthesis in the COMT line, however, did not result in relative cellulose increases of the same magnitude, but contributed to higher xylan content (12%) in the second‐year growth tissue (Figure 2, top right). Overall, for established year 2 plants, the V1 transgenic lines exhibited increased primary carbohydrate abundance, showing that recalcitrance reduction strategies can also produce plants with higher sugars. This was not the case for the miRNA transgene which does not directly target biosynthesis of cell wall components.

### Recalcitrance reductions indicated by bioconversion to ethanol

**Figure 3:**
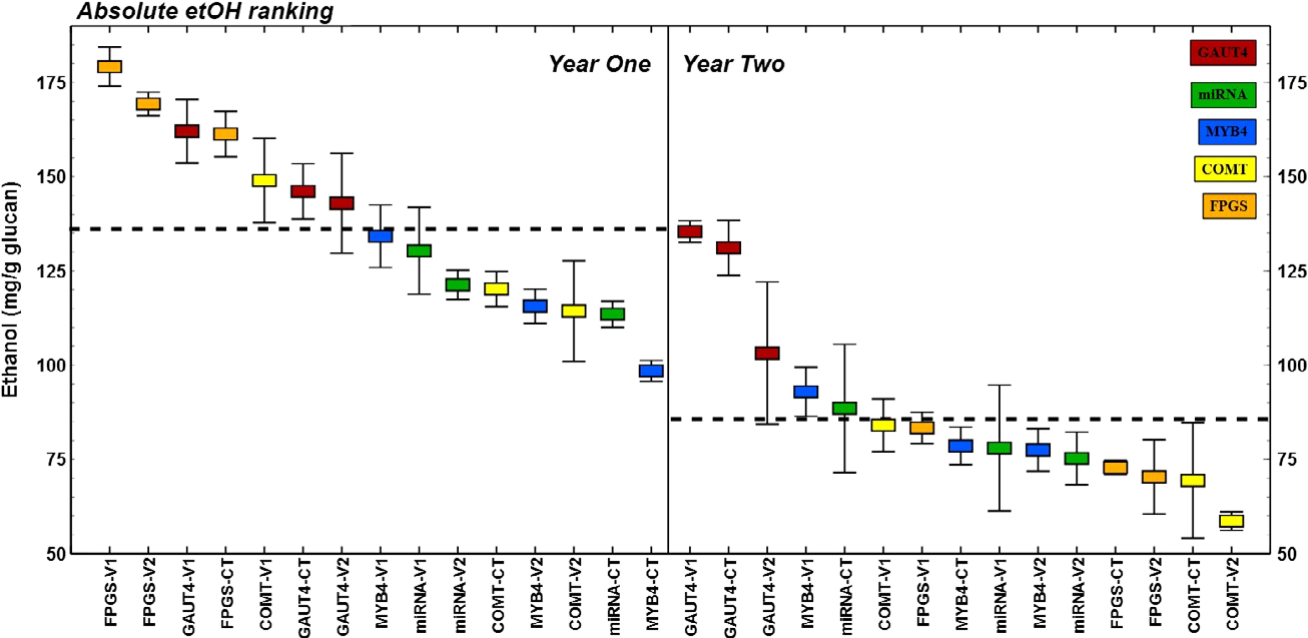
Ethanol yield (mg/g glucan) in all switchgrass lines as affected over years. Tissue hardening after regrowth from belowground biomass in the field resulted in moderate to severe increase in recalcitrance to bioconversion for all switchgrass lines. Horizontal dotted lines represent yearly averages that are significantly different at 95% confidence level.

Ethanol yield was normalized to plant glucan content for bioconversion efficiency ranking based on glucan input (mg/g glucan; Figure**** 3) and normalized to biomass used in bioconversion (mg/g biomass) for alternative ranking associated with actual feedstock input (Figure S3). The former ranking of the data was used in the analyses that follow; however, the conclusions presented here were also valid for calculations using the latter ranking.

Every transgenic and control line in this study had a sizable and significant decrease in bioconversion to ethanol from its second‐year tissue versus its first‐year tissue (Table**** 1), a trend that was also observed in the all‐plants yearly averages (*P* < 0.001 in Mann–Whitney *U*‐test; Figure 3). This was more pronounced in the ranking of ethanol yield per glucan basis due to the general increase in cellulose content of the matured year two plants (Table**** 1). The GAUT4‐CT plants recorded the smallest year‐over‐year decrease (10% relative change), while the FPGS‐V2 transgenic line had the largest decrease at 58% (Table**** 1). Reduction in bioconversion to ethanol corresponds to a broad increase in biomass recalcitrance of unknown mechanism theorized as plant tissue ‘hardening’ from environmental exposure and regrowth in the field over years from the perennial root. Data indicating this were reported for transgenic lines modifying PvMYB and COMT expression (Baxter *et al*., 2014, 2015). Our report extends this observation as a general trend affecting all transgenic and control switchgrass lines studied within BESC. This trend towards increased recalcitrance in switchgrass tissue over years was also observed in native population studies (Serba *et al*., 2016).

The leading V1 transgenic line was compared with its corresponding CT control and showed a significant increase in bioconversion to ethanol (Figure**** 2, bottom left) for the first‐year field growth, pointing to a successful reduction in plant recalcitrance for all transgenic strategies. In particular, the MYB4 modification led to a 36% ethanol yield gain. Plant hardening in the second year decreased ethanol yield gains across all transgenic lines compared with their controls, with the least effect on the COMT‐V1 transgenic construct that showed 21% gain in year 2 versus a 24% gain in the previous year. Figure 2 summarizes the overall success of the various transgene strategies for improving biomass recalcitrance. With the exception the miRNA line which targeted other aspects of plant physiology than cell wall synthesis, the mature, established V1 transgenic plants exhibited higher glucan and xylan content with lower recalcitrance to biological enzymatic solubilization compared with their respective controls. The GAUT4‐V1 transgenic plants also consistently produced higher biomass (Figure 2, bottom right). This demonstrates the overall success of transgenic switchgrass strategies for a cellulosic biofuel economy.

**Figure 4:**
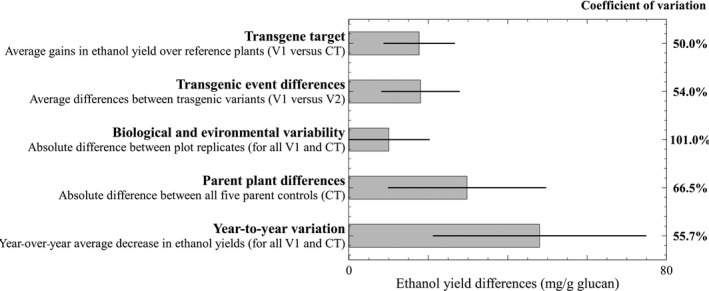
Quantitative analysis of five factors that influenced bioconversion to ethanol. Coefficients of variation describe sample spread around mean. Error bars are one standard deviation.

The different plant lines analysed over time in this study varied more than threefold in ethanol yields (Figure**** 3), from the highest ranking plant line in year one (185.9 mg/g glucan) to the lowest ranking line in year two (56.7 mg/g glucan). Multiple factors contribute to biological recalcitrance, some of which have been noted previously in this report. Recalcitrance variability was grouped into five categories and the degree of influence for each category on the bioconversion performance of these plants illustrated (Figure**** 4). These groups are not exclusive or exhaustive, but they can be numerically compared with a common metric of bioconversion to ethanol yield (mg/g glucan) and may be used as guiding elements in the analysis of future transgenic lines.

The *transgene target* group shows the average gain in ethanol yield due to transgenic modifications and is the ‘reference category’ for comparison with other groups. It considers the best transgene line (V1) for each target gene or small RNA against its respective control (and excludes the year 2 miRNA156‐V1 which did not outperform its control). The *transgenic event differences* group shows the average difference between the first (V1) and the second best (V2) independent transgenic lines for each target gene and emphasizes the importance of testing multiple gene delivery or insertion events in the plant transformation process as has been demonstrated previously (Bhat and Srinivasan, 2002; Butaye *et al*., 2005). All pairwise V1 to V2 comparisons were statistically significant at 95% confidence. *Biological and environmental variability* represents the average of absolute mean differences between plot replicates and is an unavoidable property of biological variation. From twenty independent plot sets each with three to four replicate plots, nine sets contained replication with significant variation in ANOVA tests at 95% confidence (a pairwise plot comparison within each set by Tukey test, found 22 significant differences of the 71 possible combinations). The average magnitude of plot replication differences is sufficiently high to emphasize the challenges of obtaining reproducibility in field grown plants and the potential to mask differences between transgene and control line values. It also reveals that recalcitrance varies significantly even in the same genetic background. The *parent plant differences* group evaluates bioconversion yield between all the control plant lines and shows that recalcitrance reductions can be significantly influenced by parent plant genetics irrespective of the presence of a transgene. The five control plant groups were significantly different in both growth years with *P* < 0.001 in Kruskal–Wallis tests. *Year‐to‐year variation* is the average magnitude of lowered ethanol yields due to regrowth hardening and puts into perspective the significance of this phenomenon. The coefficients of variation (Figure 4)—the spread of samples normalized to their averaged size—showed that *biological and environmental variability* was the most unpredictable and inconsistent factor impacting recalcitrance changes.

There is a power in these comparative analyses beyond the appropriate conclusions drawn from a study of a single transgenic modification. This analysis across multiple transgenic lines strongly highlights the importance of the age of the perennial plant including the original parental stock. The strong influence of the parental stock and the gains as a result of transgene expression bode well for breeders to incorporate these new low recalcitrant traits into commercial lines.

### Agronomic performance

Measurements of plant recalcitrance reductions are contingent on the bioconversion platform of choice. Furthermore, different experimental approaches may somewhat alter comparisons between lines; therefore, our efforts were centred on broader correlations and analyses such as year‐over‐year changes and collective transgene gains.

For a further understanding of the impact of each transgene strategy, the agronomic biomass data are also provided (Table 2). Assuming a single feedstock system, a 300‐million‐litre‐per‐year cellulosic ethanol plant would require 907 000 metric tons of dry feedstock per year. Furthermore, the feedstock would have to be grown within a 48‐km radius around the biorefinery to be economically sustainable, heightening the importance of the dry matter yield potential of the feedstock (Mitchell *et al*., 2008). For example, while the FPGS‐V1 plants displayed less recalcitrance, the yield of biomass in FPGS transgenics and parental controls was relatively low; therefore, potential use at industrial scale would have to also be balanced against field performance metrics. Conversely, the GAUT transgenics had less recalcitrance with higher biomass yield (as well as higher glucan and xylan content over the control line), suggesting greater potential for this line.

**Table 2:** Plant biomass yield by year of growth (averages of replicate plots; one standard deviation in parentheses). Year 1 described field establishment while year 2 showed matured regrown plants. Values represent averages from three to 10 plots, where each plot contained four clonal plants for GAUT4, miRNA, MYB4 and FPGS, and nine clonal plants for COMT. All plants increased their biomass in the second year (changes were significant at 95% confidence level with the exceptions marked with N/S superscript). For the COMT field, the aboveground biomass of all lines was cut back in August during the first growing season (Baxter *et al*., 2014), and the yields of the regrowth that occurred between September and December were not included to avoid confusion

	Biomass yield (g/plant) Year 1	Biomass yield (g/plant) Year 2	Relative change (%)
GAUT4‐V1	264 (39)	692 (159)	162
GAUT4‐V2	202 (33)	783 (76)	288
GAUT4‐CT	189 (5)	400 (66)	112
miRNA‐V1	303 (37)	968 (141)	219
miRNA‐V2	191 (23)	1245 (164)	553
miRNA‐CT	122 (37)	999 (77)	720
MYB4‐V1	54 (15)	647 (41)	1094
MYB4‐V2	38 (14)	484 (146)	1164
MYB4‐CT	54 (11)	578 (65)	981
COMT‐V1	–	575 (145)	–
COMT‐V2	–	732 (109)	–
COMT‐CT	–	745 (245)	–
FPGS‐V1	60 (5)	204 (35)	242
FPGS‐V2	36 (4)	179 (63)	^N/S^396
FPGS‐CT	66 (11)	206 (20)	213

The current study establishes precedence for the comparison of future transgenic switchgrass lines and summarizes progress and challenges with past transgene strategies. Overall, field‐grown mature transgenic V1 lines had higher glucan and xylan content and lower recalcitrance to bioconversion than parental controls. It was shown that plant hardening in field growth conditions occurred after the first regrowth from the root resulting in a drastic recalcitrance increase. Uncorrelated to recalcitrance changes, carbohydrate abundance (i.e. glucan and xylan) also increased in the regrowth process. The wealth of data and observations analysed in this study may aid in a better mechanistic understanding of plant tissue recalcitrance.

## Experimental procedures

### Switchgrass line nomenclature

All switchgrass analysed was derived from the lowland cultivar ‘Alamo’, and each transgenic experiment line had a different source of the parent lineage; therefore, variation among parent sources was to be expected. Transgene stability in the field has been verified for each experiment by expression studies. For each gene, two transgenic lines, produced from independent transformation events, were designated with suffix V1 and V2. For the GAUT4, FPGS and MYB4 lines, the parental nontransgenic control plants were clones of the plant that served as a source of tissue (explant) for transformation of the specific gene under study (the term ‘clone’ here refers to cuttings such as tillers that are then put in soil and whole plants obtained); for the COMT line, the control plants were clones derived from a line that lost the transgene through segregation in the T1 generation after cross‐hybridization of a transgenic plant with a nontransgenic ‘Alamo’ (Fu *et al*., 2011); and for the miRNA line, the control plants were clones from a plant regenerated from the ‘Alamo’ seed‐derived explant source used to produce the miRNA line. In this study, all controls were denoted with the suffix CT. The V1 label was assigned, postanalysis, to the transgenic line for each target gene or regulatory element yielding the highest ethanol yields from the second‐year analysis of each respective experiment. All lines were T0 generation except for COMT lines and their control, which were T1 generation. Clones representing each transgenic and control line were grown in three to four plot locations, and each plot contained four clonal replicates except for the COMT line, which had nine clonal replicates. All plants were grown in the same field site in Knoxville, Tennessee, over two consecutive years. Replicates were arranged in a completely randomized design in all fields except for FPGS, which was arranged in a randomized block design. Fields were maintained as described previously (Baxter *et al*., 2014). Briefly, no fertilizers or herbicides were applied for the duration of the field trail and weeding was performed by hand or tillage. Plants were irrigated as needed during the establishment year.

After each growing season (in December), the senesced aboveground biomass was harvested and dried as described previously (Baxter *et al*., 2014). Dry weight was determined after oven‐drying the harvested material at 43 °C for 168 h. Subsamples taken from each replicate were chipped into 5‐ to 8‐cm pieces and subsequently milled with a Wiley mill (Model 4; Thomas Scientific, Swedesboro, NJ) through a 1‐mm screen for downstream cell wall and fermentation analysis.

Year one represented the establishment year in the field, and year two represented established plant regrowth from belowground biomass (Table S1). Carbohydrate content data from year 3 or 4 of field growth were used in one instance in the current study, as specified, to discuss plant establishment in the field beyond the second year.

Tissues of replicate plant clones within a plot position were mixed before analyses. Therefore, for statistical analyses, biological replication of a transgenic or control line was at plot level, while technical replication per plot was carried out with duplicate or triplicate measurements, as specified.

### Microbial strain and culture media

*Saccharomyces cerevisiae* D5A (ATCC 200062) was used in SHF assays. It was maintained on YPD (10 g/L yeast extract, 20 g/L peptone and 10 g/L glucose) agar plates. The same medium was used for overnight inoculum growth.

### Carbohydrate content analysis by quantitative saccharification

Carbohydrate content of the dried (40 °C, overnight) and ground biomass solids was determined by the quantitative saccharification assay NREL/TP‐510‐42618 and high‐performance liquid chromatography (HPLC) method NREL/TP‐510‐42623. Briefly, biomass carbohydrates were acid‐hydrolysed in 72% w/w H_2_SO_4_ (0.1 g solids/mL acid) for 1 h at 30 °C, followed by further oligomer breakdown in 4% w/w H_2_SO_4_ at 120 °C for 1 h. Calcium carbonate was used to neutralize the hydrolysed samples to pH ~7. Samples were then filtered before quantification of carbohydrate content (glucose, xylose, galactose, mannose and arabinose) by HPLC (LaChrom Elite™; Hitachi High Technologies America Inc., Schaumburg, IL USA) against known standards. HPLC product separation was carried out using an Aminex™ HPX‐87P column (Bio‐Rad Laboratories Inc., Hercules, CA) at 0.6 mL/min flow rate of ultrapure water and column temperature of 80 °C, while signal was measured with a refractive index detector (model L‐2490) at 35 °C. Furfural and 5‐hydroxymethyl furfural were also quantified (UV–Vis L‐2420 detector) to confirm optimum acid solubilization with only trace amounts of monomeric sugar breakdown. Individual plot samples were analysed in triplicate technical replicates.

### Determination of recalcitrance to bioconversion: ethanol production via enzymatic hydrolysis and yeast‐based fermentation

Separate hydrolysis and fermentation of switchgrass samples were carried out in batch bottles with a 5.0% (w/v) solids loading at 20 mL final volume. In the initial hydrolysis step, the biomass was incubated with enzymes, 0.05 mL streptomycin (0.063 mg/mL final concentration) and 16 mL of ultrapure water at 50 °C for 5 days with gentle shaking. Enzymes consisted of a mixture of cellulases Cellic^®^ Ctec2, loaded at 24 FPU/g cellulose, the beta‐glucosidase Novozymes 188 and hemicellulases Cellic^®^ Htec2, which were loaded at 25% and 20% volume ratio to Ctec2, respectively. All enzymes were donated by Novozymes North America (Franklinton, NC). Aqueous samples (1 mL) were collected at the end of hydrolysis to measure sugar release and the bottles were then inoculated with a mixture of exponential growth *S. cerevisiae* D5α (2.5% v/v), citrate buffer (50 mm final concentration), yeast extract (0.5% w/v final concentration) and sterile water to a final volume of 20 mL. Sugar fermentation continued at 35 °C for 3 days with mixing, and weight loss measurements were taken at regular intervals to monitor progress. Fermentation ethanol yield was determined at endpoint by HPLC quantification using an Aminex™ HPX‐87H column (Bio‐Rad Laboratories Inc.) at 0.5 mL/min flow rate of 5 mm H_2_SO_4_ mobile phase and column temperature of 60 °C with signal quantified on a refractive index detector (model L‐2490) at 35 °C. Individual plot samples were analysed in duplicate technical replicates.

### Statistical analysis and plotting

All statistical analyses were made with Origin Pro software (OriginLab, North Hamptom MA USA) and all figures created with Veusz Scientific Plotting software (home.gna.org/veusz).

Normality of sample distributions was tested with the Shapiro–Wilk and the Kolmogorov–Smirnov tests. All samples representing a single transgenic variant or control were normally distributed, and comparisons for such samples sets were made with parametric tests (e.g. *t*‐test for comparison of two means, ANOVA for comparison of multiple sets, followed by Fisher's least significant difference test or the Tukey test). The *t*‐test was applied with equal or unequal variances based on the results of an *F*‐test for each comparison. Sample sets spanning multiple transgenic events or lines, such as yearly averages, did not respect normality (i.e. data distributions were not Gaussian). Therefore, nonparametric tests were used accordingly (e.g. Mann–Whitney *U*‐test for comparison of two means, Kruskal–Wallis for comparison of multiple sample sets).

The correlations between glucan or xylan content and ethanol yield and the correlations between year‐over‐year changes in glucan or xylan abundance and the change in ethanol yields were calculated with the Spearman test. These correlations were made across samples (at plot level) from all transgenic variants and controls and did not respect bivariate normality for Pearson linearity correlation. However, a Pearson coefficient was also calculated for guidance (although the Pearson correlation remains a consistent estimator of linearity, the test of its significance cannot be trusted without normality assumption).

Five factors that influence recalcitrance and bioconversion efficiency were defined and calculated using ethanol yield (mg/g glucan) as follows: (i) *transgene target*: the average of differences between the V1 transgenic line sample value and its respective nontransgenic control; (ii) *transgenic event differences*: the average of differences between the V1 transgenic line sample and its corresponding V2 transgenic line sample; (iii) *biological and environmental variability*: the average of absolute differences between replicate plots (e.g. four replicate plots had six possible combination differences and so on, keeping in mind that a plot had mixed plant tissue of multiple clones); (iv) *parent plant differences*: the average of absolute differences between all parent groups (i.e. five parental sources had ten combination differences); and (v) *year‐to‐year variation*: the average of all differences between year one and year two plant groups (e.g. the difference between year one COMT‐V1 and year two COMT‐V1 and so on).

Technical variation of downstream measurements is inherent in all categories, and for ethanol yield determinations by SHF with our procedure, it is under 4% and therefore considered low enough on the scale of calculations obtained in the above categories.

Error bars in all plots represent one standard deviation of the mean. The coefficient of variation (i.e. the ratio of standard deviation to the mean) is a measure of sample dispersion.

## Supporting information


**Figure S1** Glucan abundance in the COMT‐V1 transgenic line and its null segregant line over four years of field growth.
**Figure S2** No correlations were found between glucan content and ethanol yield and between year‐over‐year changes in plant glucan abundance and changes in ethanol yield.
**Figure S3** Decreasing rank of ethanol yield (mg/g biomass) separated by year of growth.
**Table S1** Transgenic and control lines with manuscript label, years of growth, original transgenic event and plot# label used in primary plant line publications (see in‐text references); and plant biomass yields averaged at plot level
