# Supplementary material for: Transgenic switchgrass (Panicum virgatum L.) targeted for reduced recalcitrance to bioconversion: a 2‐year comparative analysis of field‐grown lines modified for target gene or genetic element expression
Source: Plant Biotechnol J. 2017 Feb 20;15(6):688–97. doi: 10.1111/pbi.12666 (PMC5425389; doi:10.1111/pbi.12666)
Supplement: Supplementary file 1 — Figure S1 Glucan abundance in the COMT‐V1 transgenic line and its null segregant line over four years of field growth. Figure S2 No correlations were found between glucan content and ethanol yield and between year‐over‐year changes in plant glucan abundance and changes in ethanol yield. Figure S3 Decreasing rank of ethanol yield (mg/g biomass) separated by year of growth. Table S1 Transgenic and control lines with manuscript label, years of growth, original transgenic event and plot# label used in primary plant line publications (see in‐text references); and plant biomass yields averaged at plot level [file PBI-15-688-s001.pdf]

Supplemental figures

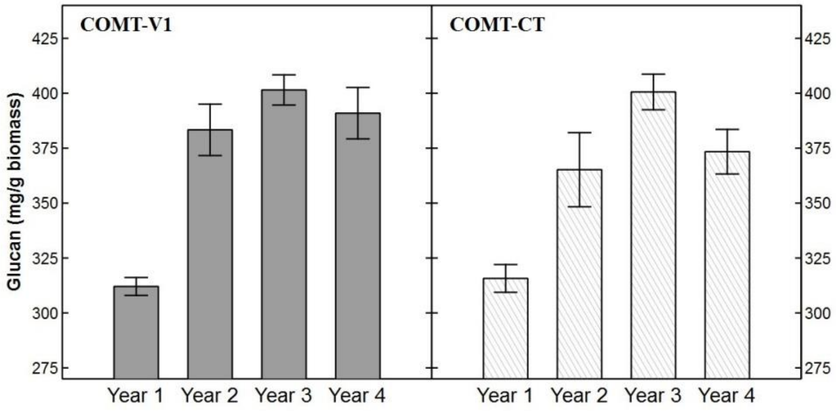

Figure A. 1. Glucan abundance in the COMT-V1 transgenic line and its null segregant line over four years of field growth. Glucan content significantly increased on the first regrowth (year 1 to year 2, at 95% confidence level) for both lines, and stabilized with smaller, yet still statistically significant differences through subsequent years (years 2, 3 and 4 compared in ANOVA test with 95% confidence level; a Fisher LSD test revealed years 2 and 4 were different from year 3 at 95% confidence level for both lines).

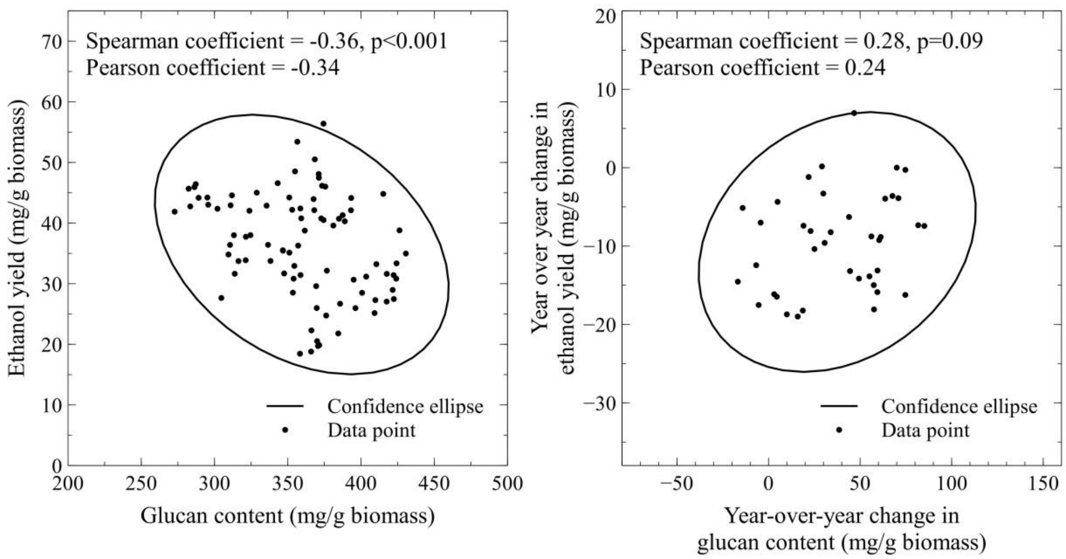

Figure A. 2. No correlations were found between glucan content and ethanol yield and between year-over-year changes in plant glucan abundance and changes in ethanol yield. Although the measurements compared were not strictly bivariate normal but were continuous, the Pearson linear correlation was also calculated and the confidence elipse of the regression was included as guidance. In theory, the elipse delimits the prediction of new measurements at 95% confidence level (i.e., a 2D confidence limit).

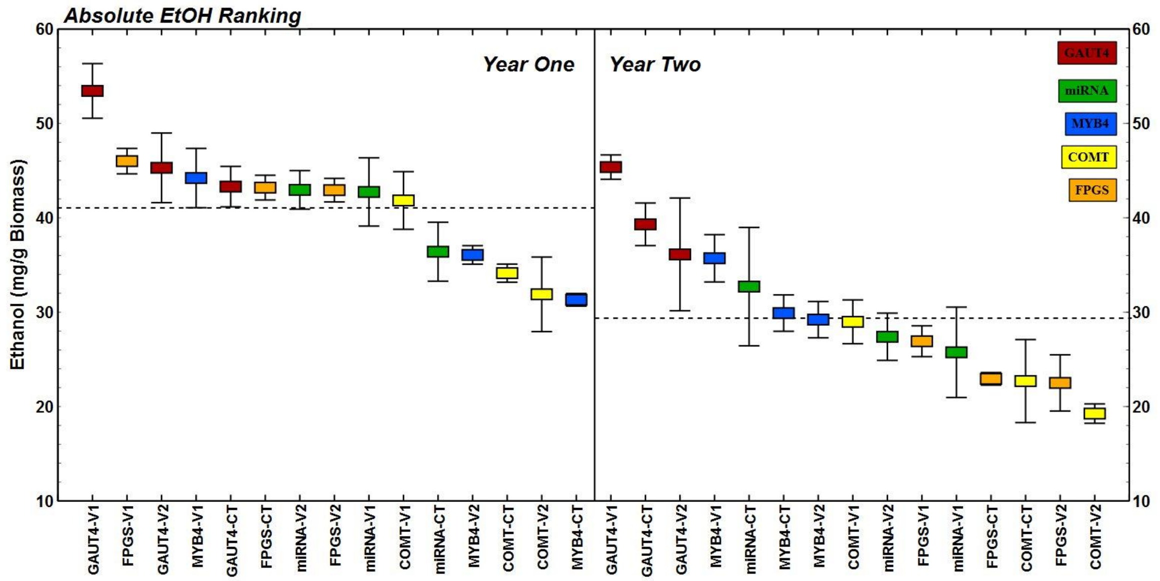

Figure A. 3. Decreasing rank of ethanol yield (mg/g biomass) separated by year of growth. All switchgrass lines recorded a year-over-year decrease in bioconversion to ethanol. The yield normalized to biomass weight was largely unchanged from its normalization to plant glucan content and shows the low correlation between carbohydrate content and conversion yields.

Table A. 1. Transgenic and control lines with manuscript label, years of growth, original transgenic event and plot# label used in primary plant line publications (see in-text references); and plant biomass yields averaged at plot level.

Manuscript name	Growth Years (Y1-Y2)	Trangenic Event (original name)	Plot # (original name)	Y1 biomass yield (g/plant)	Y2 biomass yield (g/plant)
GAUT4-V1	2013, 2014	4A	4	240.0	875.0
			9	244.0	600.0
			13	309.5	600.0
GAUT4-V2	2013, 2014	2B	1	230.0	850.0
			5	165.5	700.0
			8	211.0	800.0
GAUT4-WT	2013, 2014	WT	2	183.0	425.0
			3	193.5	450.0
			12	190.5	325.0
miRNA-V1	2013, 2014	T37	10	314.5	940.0
			11	314.0	792.5
			15	335.0	1129.5
			17	250.0	1010.0
miRNA-V2	2013, 2014	T35	2	166.0	1016.5
			9	208.5	1301.0
			16	176.5	1256.5
			20	211.5	1404.5
miRNA-WT	2013, 2014	WT	3	77.0	927.0
			8	147.5	1033.5
			12	106.5	945.5
			14	156.5	1090.5
MYB4-V1	2012, 2013	L8	22	71.0	618.0
			38	43.3	693.5
			42	48.1	628.5
MYB4-V2	2012, 2013	L6	27	51.4	496.0
			33	23.8	332.0
			37	39.6	623.0
MYB4-WT	2012, 2013	L10	26	41.5	575.5
			31	63.8	644.0
			39	55.1	515.0
COMT-V1	2011, 2012	COMT3-NF01	15	13.1	741.3
		COMT3-NF09	2	12.0	510.0

		COMT3-NF03	1	8.1	474.4
COMT-V2	2011, 2012	COMT2-NF12	7	9.8	688.3
		COMT2-NF06	4	11.9	856.7
		COMT2-NF09	28	13.3	652.2
COMT-WT	2011, 2012	COMT3-NF12	17	23.9	552.2
(COMT3-null segregant)		COMT3-NF14	26	25.0	1021.1
		COMT3-NF15	29	20.8	661.7
FPGS-V1	2014, 2015	T10	7	60.0	182.5
			9	54.5	245.0
			17	64.5	185.0
FPGS-V2	2014, 2015	T2	1	37.0	110.0
			13	31.5	233.3
			20	39.5	192.5
FPGS-WT	2014, 2015	WT	6	59.5	183.3
			10	78.5	220.0
			18	59.5	215.0
NOT USED IN THE MANUSCRIPT					
COMT2-null segregant	2011, 2012	COMT2-NF04	3	8.9	525.0
		COMT2-NF03	11	7.1	541.1
		COMT2-NF05	24	24.0	699.4
